# Electrochemical Synthesis and Electro-Optical Properties of Dibenzothiophene/Thiophene Conjugated Polymers With Stepwise Enhanced Conjugation Lengths

**DOI:** 10.3389/fchem.2020.00819

**Published:** 2020-09-08

**Authors:** Kaiwen Lin, Caiting Li, Wang Tao, Jilong Huang, Qinghua Wu, Zijin Liu, Yangfan Zhang, Da Wang, Xi Liu

**Affiliations:** ^1^Department of Materials and Food, Zhongshan Institute, University of Electronic Science and Technology of China, Zhongshan, China; ^2^School of Textile Materials and Engineering, Wuyi University, Jiangmen, China; ^3^School of Applied Physics and Materials, Wuyi University, Jiangmen, China

**Keywords:** dibenzothiophene, electrochemical synthesis, electrochromism, conjugation lengths, color changes, electrochemical stability

## Abstract

A total of six conjugated polymers, namely PDBT-Th, PDBT-Th:Th, PDBT-2Th, PDBT-Th:2Th, PDBT-2Th:Th, and PDBT-2Th:2Th, consisting of dibenzothiophene, thiophene, and bithiophene were electrochemically synthesized. Their electrochemical and electrochromic properties were investigated in relation to the conjugation chain lengths of the thiophene units in the conjugated backbones. Density functional theory (DFT) calculations showed that longer conjugation lengths resulted in decreased HOMO-LUMO gaps in the polymers. The optical band gaps (*E*_g,opt_) and electrochemical band gaps (*E*_g,cv_) were decreased from PDBT-Th to PDBT-Th:Th, however, PDBT-Th:2Th, PDBT-2Th, PDBT-2Th:Th and PDBT-2Th:2Th displayed the similar band gaps. The conjugation length increments significantly improved the electrochemical stability of the conjugated polymers and exhibited reversible color changes due to the formation of polarons and bipolarons. The results suggest that the conjugated polymers prepared herein are promising candidates for fabricating flexible organic electrochromic devices.

## Introduction

It is indispensable for the preparation of high-performance conjugated polymers and development of state-of-the-art applications to explore the structure-performance relationship of conjugated polymers. Breakthroughs in organic optoelectronics, including organic solar cells, dye-sensitized solar cells, organic field effect transistors, and electrochromism (Zhang et al., 2013; Jin et al., 2014; Zhou et al., 2015; Li et al., 2019a), has been performed by altering the main conjugation length. Strategies for altering the main conjugation length of organic optoelectronic materials include chemical and electrochemical polymerization methods (Jin et al., 2014; Zhou et al., 2015).

Electrochemical polymerization is often used in organic optoelectronics and employs a working electrode on which the polymer films simultaneous polymerize and are deposited by an applied voltage (Li et al., 2009; Jiang et al., 2018). Electrochemical polymer preparation exhibits several advantages: (1) It is high-throughput for synthesizing polymer films, formed only on the electrodes without any observed in solution; (2) It is highly efficient for synthesizing polymer films, since the reaction can be conducted in several seconds or minutes, while conventional chemical synthesis of a similar polymer requires several hours or days; (3) The electropolymerization method uses cheap supporting electrolytes instead of specific catalysts and expensive complexants for solution-phase synthesis; (4) The reaction can be performed at room temperature, whereas conventional chemical synthesis usually requires harsh conditions with high temperatures under an inert atmosphere (Gu et al., 2015; Yuan and Lei, 2020). The extension of the main chain conjugation length *via* electrochemical polymerization is effectively concentrated in electrochromism (Ak et al., 2008; Camurlu et al., 2008; Kavak et al., 2015; Sheberla et al., 2015; Gu et al., 2018; Li et al., 2019b; Zhang et al., 2019; Lu et al., 2020). Electrochromism is often defined as the visible and reversible changes in the transmittance and color of a material caused by an applied voltage (Argun et al., 2004; Beaujuge and Reynolds, 2010; Lin et al., 2017). For example, Zhao et al., verified the stepwise enhancement of the electrochemical and electrochromic performances of polyselenophene *via* electropolymerization of mono-, bi-, and triselenophene. Polyselenophene that was electropolymerized from triselenophene exhibited the lowest optical band gap (1.72 eV), highest redox stability, as well as the best electrochromic nature of optical contrast up to 75%, coloration efficiency up to 450 cm^2^ C^−1^, and switching time (0.7 and 0.4 s for oxidation and reduction, respectively) compared to polyselenophene prepared from mono- and biselenophene (Lu et al., 2020). Zhang et al., prepared a cross-linked copolymer (pTPhSNS-EDOT) *via* electrochemical polymerization that exhibited a fast coloring time of 0.58 s and discoloring time of 0.38 s, high optical contrast of 40%, excellent color stability, and improved color memory behavior compared to the pTPhSNS homopolymer (Dai et al., 2017). Therefore, extension of the main chain conjugation length is beneficial for obtaining excellent electrochemical and electrochromic properties.

Herein, thiophene, and thiophene derivatives were used to construct electrochromic polymers with stepwise enhancement of the main chain conjugation lengths *via* electrochemical copolymerization. The relationship between the main chain conjugation length and electrochromic properties, as well as the electrochemical redox activity and stability of the conjugated polymer, were studied in detail. Significantly, this study provides theoretical guidance for the development of related fields.

## Result and Discussion

### Synthesis and Characterization

**Scheme 1:**
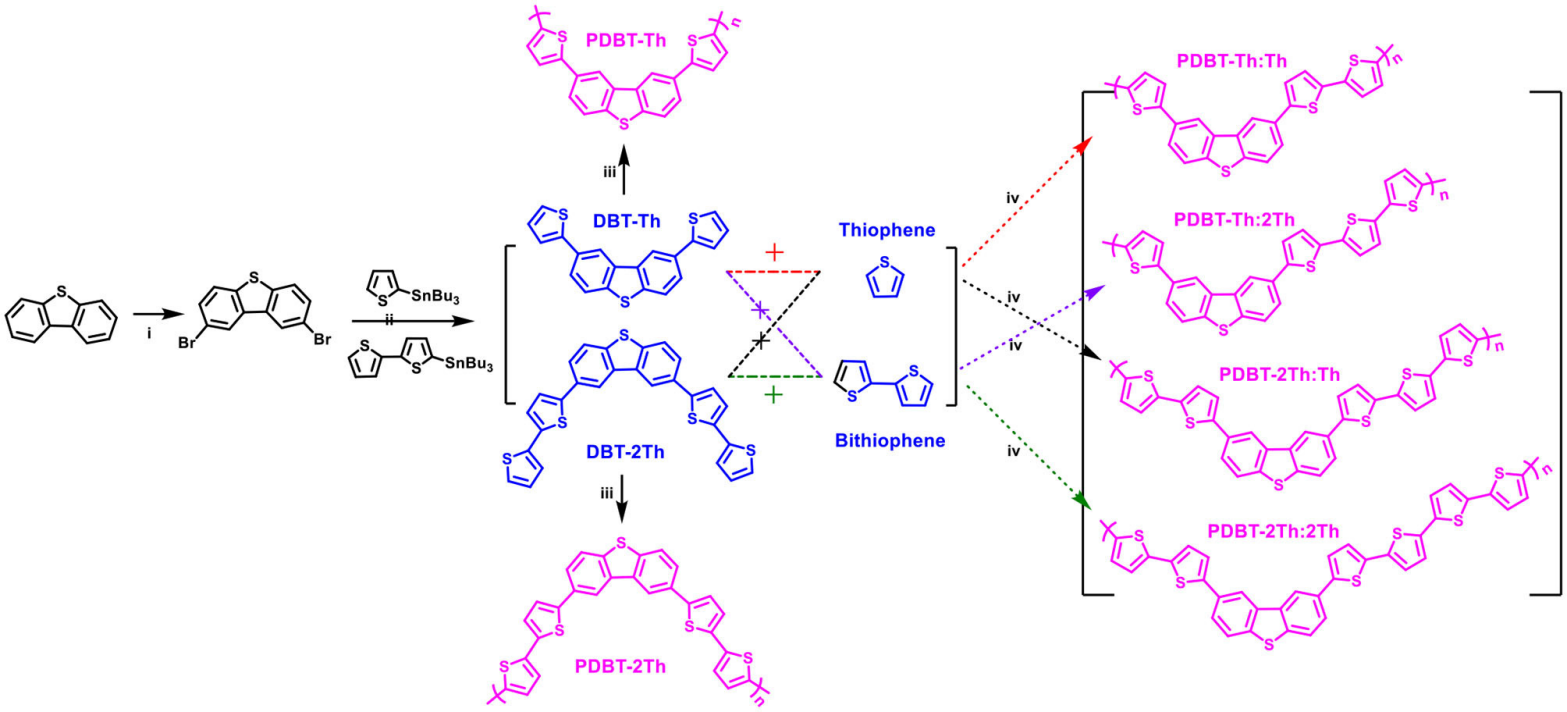
Synthetic routes of monomers (DBT-Th and DBT-2Th) and electrochemical polymers (PDBT-Th, PDBT-2Th, PDBT-Th:Th, PDBT-Th:2Th, PDBT-2Th:Th, and PDBT-2Th:2Th). Reagents and conditions: (i) CHCl_3_, Br_2_ (2.2 eq.), N_2_ (ii) Pd(PPh_3_)_4_, chlorobenzene, 90°C, (iii) electrochemical polymerization, CH_2_Cl_2_-Bu_4_NPF_6_, (iv) electrochemical copolymerization, CH_2_Cl_2_-Bu_4_NPF_6_.

The synthetic routes for the monomers (DBT-Th and DBT-2Th) and electrochemical polymers (PDBT-Th, PDBT-2Th, PDBT-Th:Th, PDBT-Th:2Th, PDBT-2Th:Th, and PDBT-2Th:2Th) are illustrated in Scheme 1. Poly[2,8-bis-(thiophen-2-yl)-dibenzothiophene] (PDBT-Th) and poly[2,8-Bis-(bithiophen-2-yl)-dibenzothiophene] (PDBT-2Th) were prepared from the 2,8-bis-(thiophen-2-yl)-dibenzothiophene (DBT-Th) and 2,8-Bis-(bithiophen-2-yl)-dibenzothiophene (DBT-2Th) monomers (0.01 mol L^−1^) and Bu_4_NPF_6_ (0.1 mol L^−1^) in DCM *via* electrochemical polymerization. PDBT-Th:Th and PDBT-Th:2Th were electrochemical polymerized from 0.005 mol L^−1^ DBT-Th and 0.005 mol L^−1^ thiophene (Th)/bithiophene (2Th), respectively. PDBT-2Th:Th and PDBT-2Th:2Th were obtained from 0.005 mol L^−1^ DBT-2Th and 0.005 mol L^−1^ Th/2Th *via* electrochemical copolymerization. From the synthetic routes of DBT-Th and DBT-2Th, dibenzothiophene (DBT) was used to prepare the corresponding bromide (2,8-dibromodibenzothiophene) *via* bromination. The above dibromide was reacted with tributyltin substituted Th/2Th to afford the target products using a Pd(PPh_3_)_4_ catalyst. The ^1^H NMR and ^13^H NMR spectra of target monomers are presented in Figures S1–S4.

### Electrochemical Polymerization of DBT-Th, DBT-2Th and Electrochemical Copolymerization of DBT-Th, DBT-2Th, Th, and 2Th

From their anodic oxidation curves (Figure S5), the onset oxidation potentials (*E*_onset_) were initiated at 1.11, 1.30, 0.85, 1.27, 1.07, and 0.98 V for DBT-Th, DBT-Th:Th, DBT-2Th, DBT-Th:2Th, DBT-2Th:Th, and DBT-2Th:2Th, respectively. The polymers were prepared by the potentiostatic method (adding about 0.2 V of *E*_onset_) along with similar charge of about 5 mC. In this electrochemical deposition condition, the thickness of polymer films were about 200 nm, which is in agreement with the reported research (Lin et al., 2020b).

**Figure 1:**
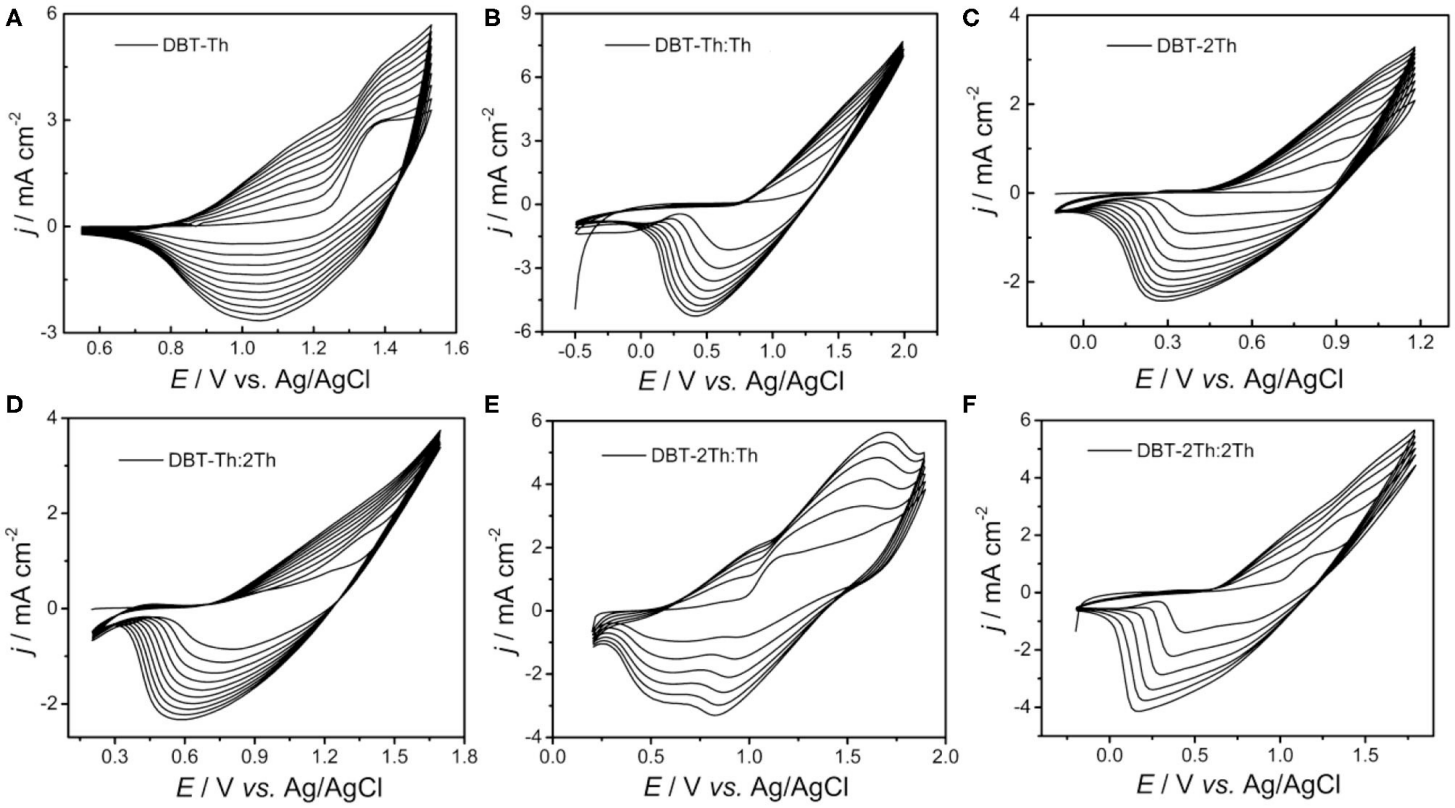
Cyclic voltammograms of DBT-Th **(A)**, DBT-Th: Th **(B)**, DBT-2Th **(C)**, DBT-Th: 2Th **(D)**, DBT-2Th: Th **(E)**, and DBT-2Th: 2Th **(F)** in CH_2_Cl_2_-Bu_4_NPF_6_. Potential scan rate: 100 mV s^−1^.

Figure 1 shows the obtained cyclic voltammograms (CVs) corresponding to the potentiodynamic electropolymerization of the polymeric precursor monomers DBT-Th and DBT-2Th as well as the potentiodynamic electrochemical copolymerization of the polymeric precursor comonomers DBT-Th, DBT-2Th, Th, and 2Th. The current density increased gradually as a function of scanning cycles for all systems. Meanwhile, the corresponding conducting polymers on the working electrode grew prominently, indicating that the as-prepared conducting polymers exhibited good electrochemical redox activity. Meanwhile, all systems exhibited an increasingly obvious voltage drop (ΔV) from 0.6 to 1.0 V of the reduction peak with stepwise enhanced conjugation lengths, which were ascribed to the wide distribution of polymer chain lengths. Because of the additional potential required to balance the increased polymeric electrical resistance and slow mass transport, an obvious potential shift of the anodic and cathodic peaks was observed during polymer growth (Chen and Xue, 2005; Lin et al., 2020a; Lu et al., 2020).

### Theoretical Calculations

**Figure 2:**
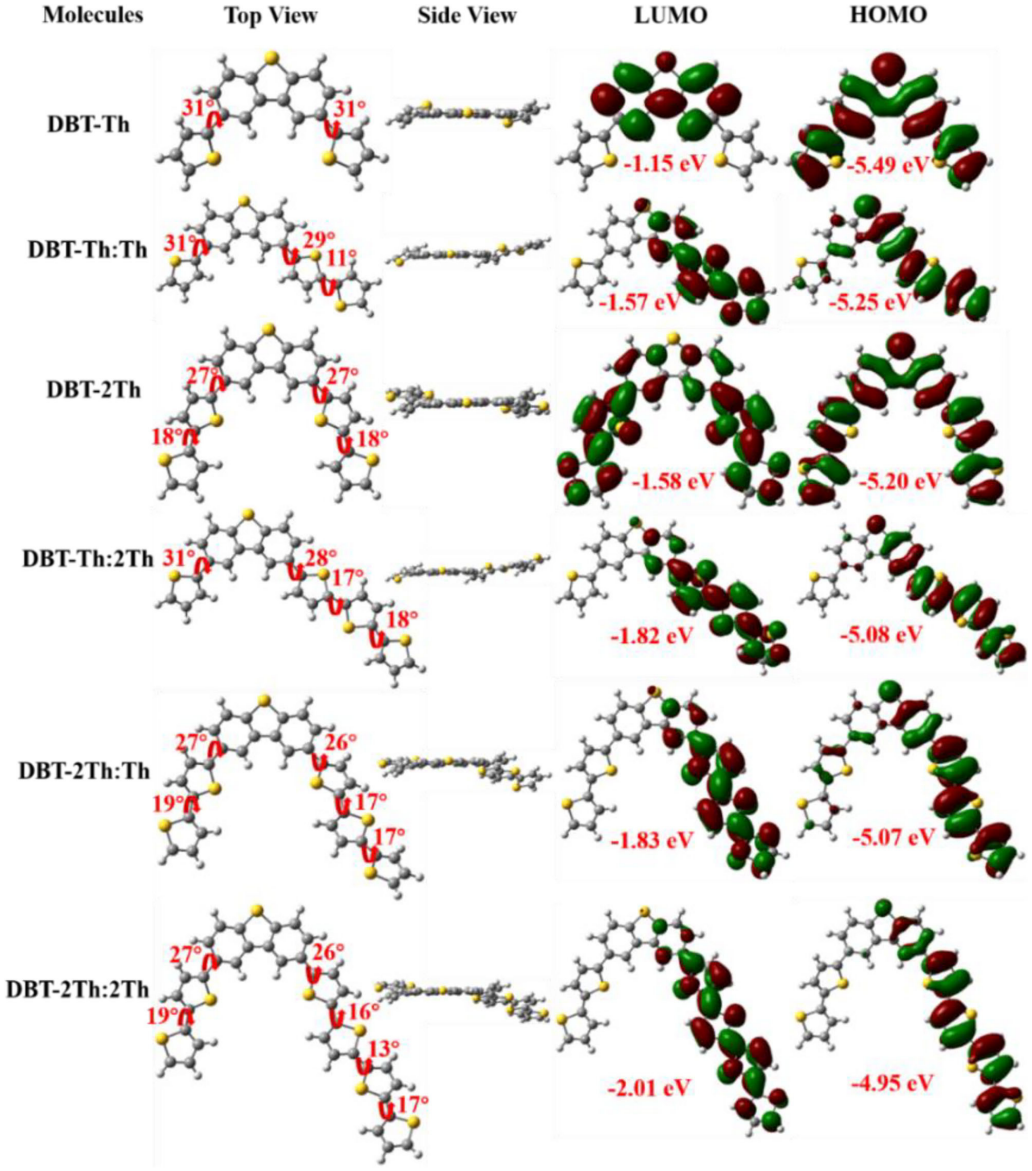
Optimized molecular geometries and frontier molecular orbital distributions of DBT-Th, DBT-Th:Th, DBT-2Th, DBT-Th:2Th, DBT-2Th:Th, and DBT-2Th:2Th obtained from DFT by Gaussian 09 at the B3LYP/6-31G(d,p) level.

The ground-state optimized molecular geometries and frontier molecular orbital distributions of DBT-Th, DBT-Th:Th, DBT-2Th, DBT-Th:2Th, DBT-2Th:Th, and DBT-2Th:2Th were determined using density functional theory (DFT) by Gaussian 09 at the B3LYP/6-31G(d,p) level (Figure 2). All optimized molecular geometries exhibited slightly twisted, n-shaped structures, with dihedral angles of <31° owing to the steric hindrance effect. The dihedral angles of DBT and adjacent thiophene decreased with enhanced conjugation lengths, which resulted in improved regularity. For all models, the electron density distribution of the lowest unoccupied molecular orbitals (LUMOs) and highest occupied molecular orbitals (HOMOs) were localized in the sectional conjugated skeleton. The longer the conjugation lengths resulted in lower energy LUMO and HOMO levels (Liu et al., 2017). The HOMO-LUMO gaps of all models gradually decreased from 4.34 to 2.94 eV with stepwise enhancement of the conjugation lengths.

### Electrochemistry of the Polymers

**Figure 3:**
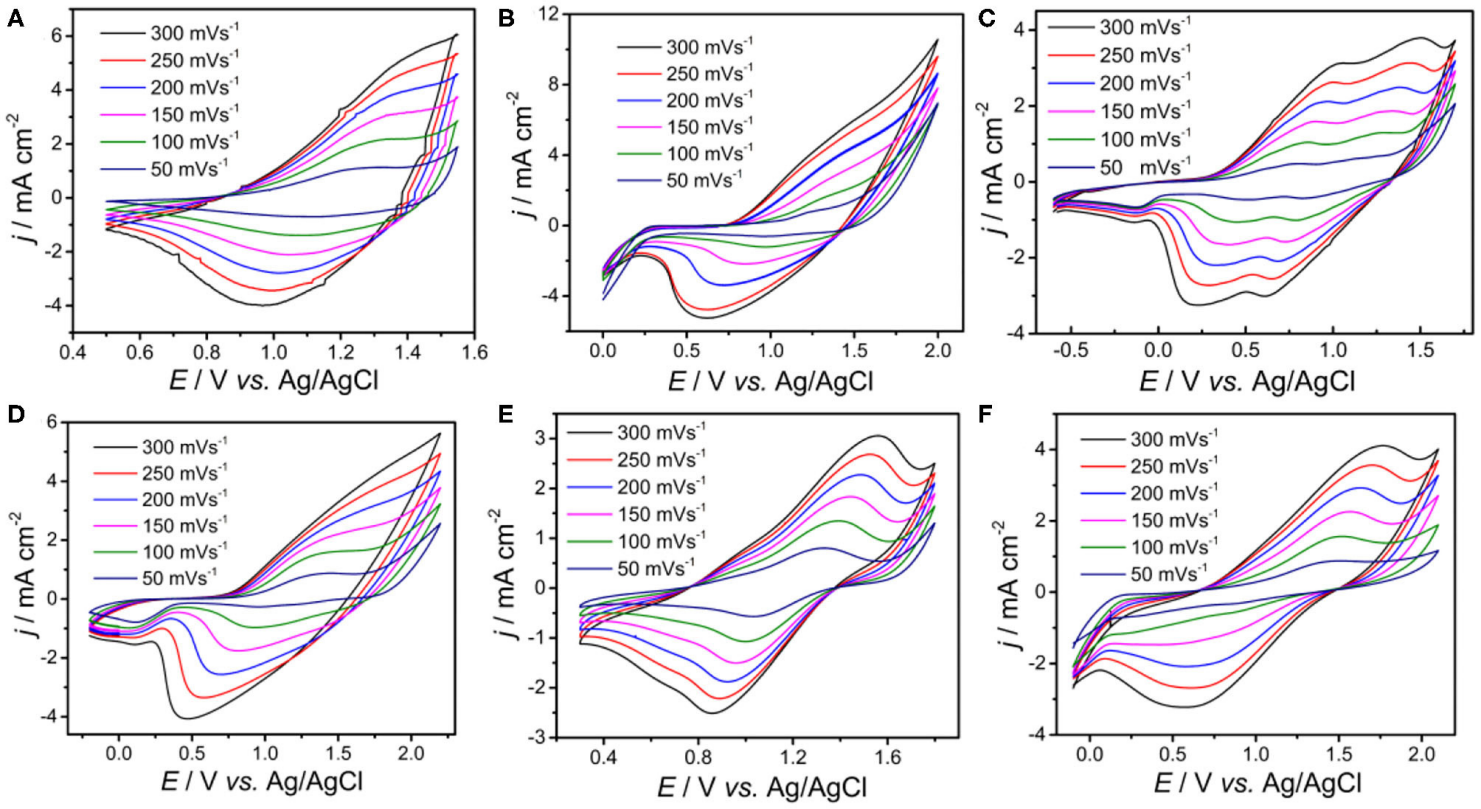
Cyclic voltammograms of the electrochemical polymer PDBT-Th **(A)**, PDBT-Th:Th **(B)**, PDBT-2Th **(C)**, PDBT-Th:2Th **(D)**, PDBT-2Th:Th **(E)**, and PDBT-2Th:2Th **(F)** modified Pt electrodes in monomer-free CH_2_Cl_2_-Bu_4_NPF_6_ (0.10 mol L^−1^) at potential scan rates of 300, 250, 200, 150, 100, and 50 mV s^−1^.

To obtain deeper insight of the electrochemical activity, the electrochemical behaviors of PDBT-Th, PDBT-Th:Th, PDBT-2Th, PDBT-Th:2Th, PDBT-2Th:Th, and PDBT-2Th:2Th were investigated *via* CVs in monomer-free CH_2_Cl_2_-Bu_4_NPF_6_ (0.1 mol L^−1^; Figure 3). All polymers were prepared using the potentiostatic method at a constant potential of 1.30 V for DBT-Th, 1.50 V for DBT-Th:Th, 1.05 V for DBT-2Th, 1.45 V for DBT-Th:2Th, 1.3 V for DBT-2Th:Th, and 1.2 V for DBT-2Th:2Th. All polymers showed obvious redox peaks with hysteresis (potential drift) between the anodic and cathodic peak potentials. The potential shifts of the redox peaks among the CVs were attributed to slow heterogeneous electron transfer, local rearrangement of the polymer chains, slow mutual transformation of various electronic species, and electronic charging of the interfacial exchange at the metal/polymer and polymer/solution interfaces (Inzelt et al., 2000).

In addition, the cyclic voltammetry was employed to evaluate the experimental HOMO and LUMO energy levels of polymers through the empirical Equations (1) and (2) in the Supporting Information (Sun et al., 2011). Meanwhile, the theoretical calculated HOMO/LUMO energy levels of polymers (simplified by two repeating units) were illustrated comparatively. The values were presented in Table 1. The experimental and theoretical calculated HOMO both exhibited up lifted values when the conjugation length was increased. The HOMO-LUMO gaps by theoretical calculation decreased gradually. The optical band gaps (*E*_g,opt_) and electrochemical band gaps (*E*_g,cv_) were decreased from PDBT-Th to PDBT-Th:Th, however, PDBT-Th:2Th, PDBT-2Th, PDBT-2Th:Th and PDBT-2Th:2Th displayed the similar band gaps.

**Table 1:** Electrochemical, optical properties and theoretical calculation of polymers.

**Polymers**	***E*_**ox,onset**_ (V)**	***E*_**red,onset**_ (V)**	**HOMO**		**LUMO**		***E*_**g,cv**_ (eV)**	**HOMO-LUMO gaps (eV)**	***E*_**g,opt**_ (eV)**
			**Experimental value**	**Theoretical value**	**Experimental value**	**Theoretical value**			
PDBT-Th	0.88	−1.95	−5.68	−5.10	−2.85	−1.73	2.83	3.37	2.59
PDBT-Th:Th	0.70	−1.75	−5.50	−5.00	−3.05	−1.97	2.45	3.03	2.53
PDBT-2Th	0.55	−1.74	−5.35	−4.90	−3.06	−2.09	2.29	2.81	2.25
PDBT-Th:2Th	0.56	−1.72	−5.36	−4.92	−3.08	−2.10	2.28	2.82	2.29
PDBT-2Th:Th	0.54	−1.75	5.34	−4.84	−3.05	−2.19	2.29	2.65	2.24
PDBT-2Th:2Th	0.52	−1.77	−5.32	−4.81	−3.03	−2.25	2.29	2.56	2.23

**Figure 4:**
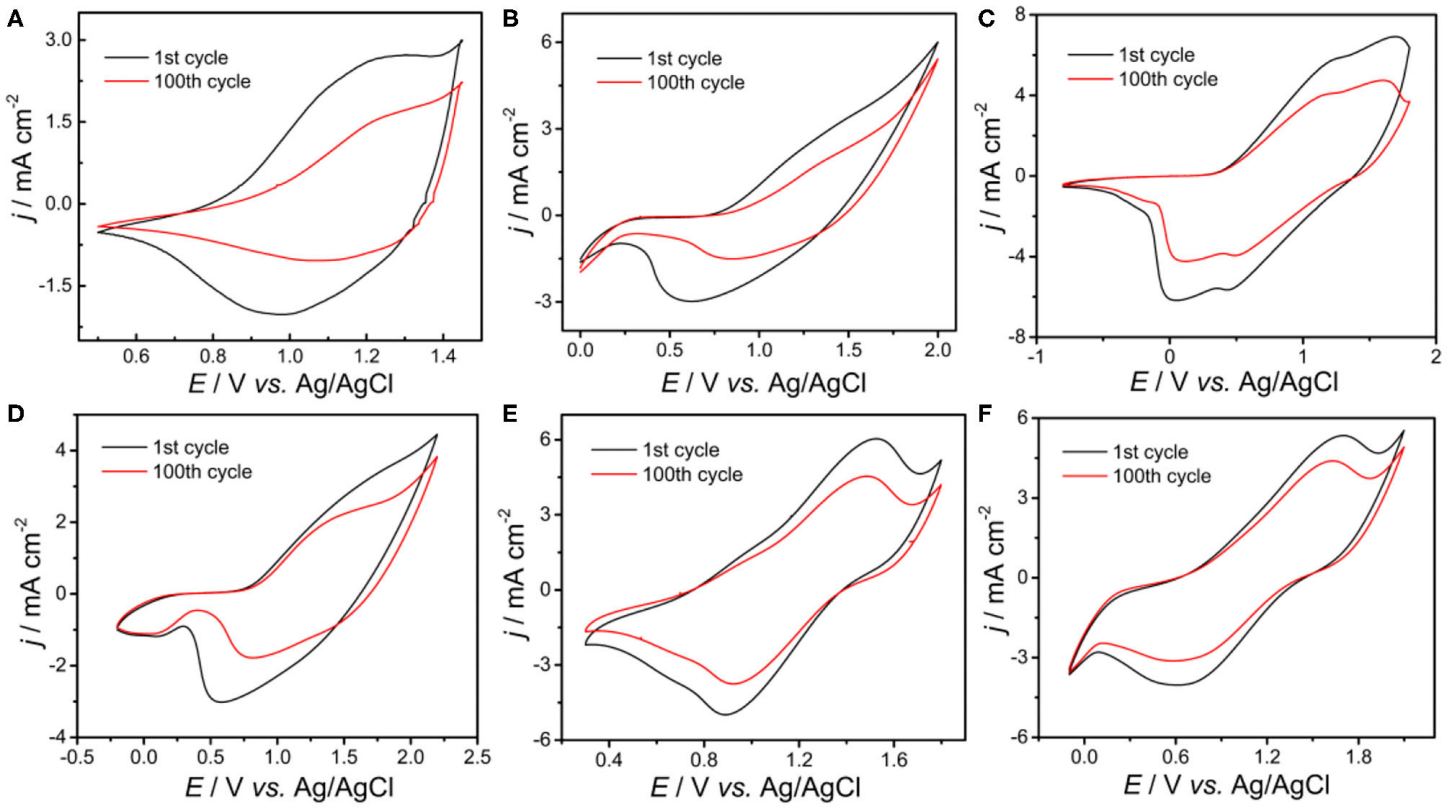
CVs of PDBT-Th **(A)**, PDBT-Th:Th **(B)**, PDBT-2Th **(C)**, PDBT-Th:2Th **(D)**, PDBT-2Th:Th **(E)**, and PDBT-2Th:2Th **(F)** cycled 100 times at a potential scan rate of 200 mV s^−1^ in monomer-free CH_2_Cl_2_-Bu_4_NPF_6_ (0.10 mol L^−1^).

The stabilities of all polymers were studied in a monomer-free CH_2_Cl_2_-Bu_4_NPF_6_ system. A total of 100 cycles were performed to study the doping and dedoping abilities of all polymers, as shown in Figure 4. The redox activity of PDBT-Th was maintained at 56% after scanning 100 cycles, exhibiting generally good redox stability. However, increasing conjugation length significantly improved electrochemical stability, achieving moderate stability at 62, 79, 76, 85, and 90% remaining activity for PDBT-Th:Th, PDBT-2Th, PDBT-Th:2Th, PDBT-2Th:Th, and PDBT-2Th:2Th, respectively, after scanning 100 cycles. The improved electrochemical stability could benefit from the more stable thiophene bridge.

### Electrochromic Properties

**Figure 5:**
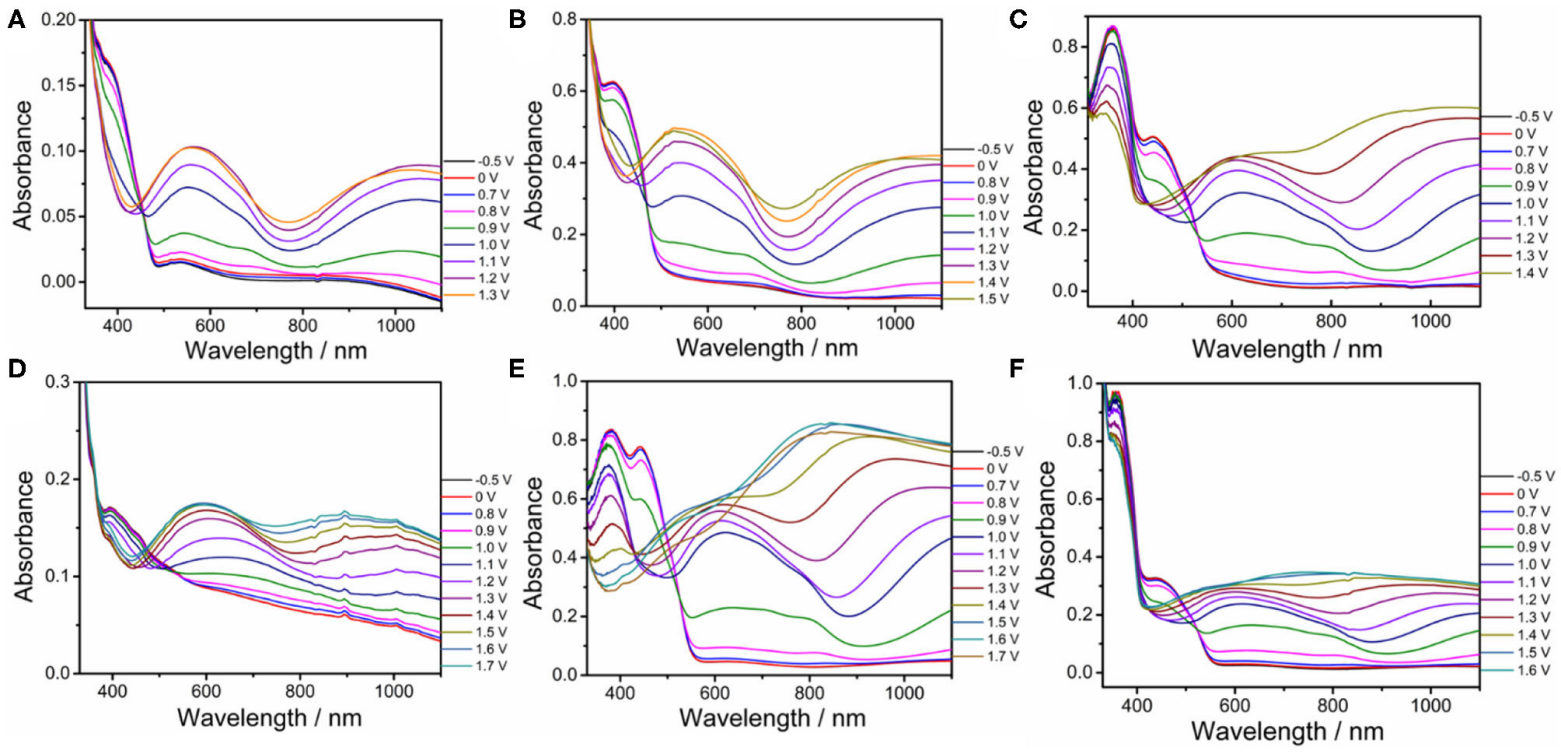
Spectroelectrochemical traces for PDBT-Th **(A)**, PDBT-Th:Th **(B)**, PDBT-2Th **(C)**, PDBT-Th:2Th **(D)**, PDBT-2Th:Th **(E)**, and PDBT-2Th:2Th **(F)** on ITO coated glass in CH_3_CN-Bu_4_NPF_6_ (0.1 mol L^−1^).

Spectroelectrochemical analyses were performed by recording the absorption changes of the polymers under different potentials (Figure 5). In the neutral state, all polymers showed the absorbance peaks centered at approximately 410 nm arising from the π-π^*^ transition. DBT-2Th based polymers PDBT-2Th (c), PDBT-2Th:Th (e), and PDBT-2Th:2Th (f) clearly showed two absorbance peaks with red shift on the absorbance edges, which was ascribed to the enhanced conjugation lengths. The optical band gaps (Table 1, empirical Equation (3) in the Supporting Information) of the corresponding polymers were gradually reduced from 2.59 eV (PDBT-Th) to 2.53 eV (PDBT-Th:Th), 2.29 eV (PDBT-Th:2Th), 2.25 eV (PDBT-2Th), 2.24 eV (PDBT-2Th:Th), and 2.23 eV (PDBT-2Th:2Th). With increasing effective conjugated chain length of those polymers by introducing thiophene as bridge unit, the optical band gaps were gradually reduced, and tend to the same. Notably, with increasing voltage, new bands resulting from the polaron at approximately 600 nm and bipolaron at approximately 1,000 nm increased in intensity (Zhu et al., 2009; Lu et al., 2014; Yang et al., 2014; Lin et al., 2015; Ming et al., 2020). During this process, all polymers displayed a conspicuous color change under doped (oxidized) and dedoped (neutral) conditions (Table 2). To determine the color change, the CIE 1976 (L^*^, a^*^, b^*^) color space and photographs were determined, in which L^*^ is the parameter of the lightness, a^*^ is the red-green balance and b^*^ is yellow-blue balance (–a^*^ and +a^*^ correspond to green and red and –b^*^ and +b^*^ correspond to blue and yellow, respectively) (Dyer et al., 2011). As presented in Table 2, PDBT-Th, PDBT-Th:Th, PDBT-2Th, and PDBT-Th:2Th showed –a^*^ and +b^*^ directions in the neutral state, therefore, the polymers exhibited yellow green color. PDBT-2Th:Th and PDBT-2Th:2Th exhibited noteworthy color of claybank (neutral state), which was attributed to the value of +a^*^ and +b^*^ directions. Isosbestic points at 450 nm of DBT-Th based polymers and 500 nm of DBT-2Th based polymers appeared in the spectra, indicating that these polymers were easily interconverted between neutral and oxidized states (Balan et al., 2011; Lin et al., 2020b).

**Table 2:** Colorimetric parameters for the prepared polymers.

**Polymers**	**CIE color coordinates**			**Colors of polymers**	
		**Neutral**	**Oxidized**	**Neutral**	**Oxidized**
PDBT-Th	L^*^	98.7235	92.2886	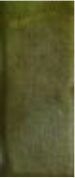	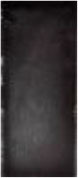
	a^*^	−2.2272	1.6750		
	b^*^	6.5666	−3.7137		
PDBT-Th:Th	L^*^	87.0708	67.1116	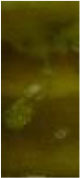	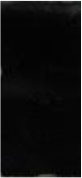
	a^*^	−8.4870	4.7474		
	b^*^	32.6390	−3.4195		
PDBT-2Th	L^*^	82.3156	79.7525	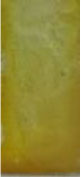	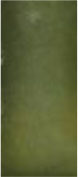
	a^*^	−1.3602	−4.6198		
	b^*^	20.1580	4.5149		
PDBT-Th:2Th	L^*^	98.8370	94.9152	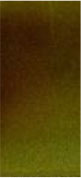	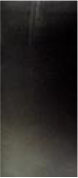
	a^*^	−0.2581	−0.1172		
	b^*^	5.9035	−4.5872		
PDBT-2Th:Th	L^*^	84.1909	69.1127	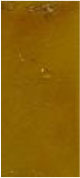	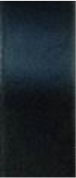
	a^*^	11.1086	−2.1452		
	b^*^	63.0452	−9.6235		
PDBT-2Th:2Th	L^*^	88.6797	76.6157	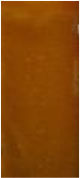	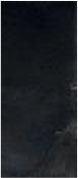
	a^*^	6.0639	−1.6808		
	b^*^	33.9582	−4.6985		

## Conclusion

Thiophene was used as a fundamental unit to progressively construct conjugated polymers of various lengths by electrochemical polymerization from polymeric precursor monomers (DBT-Th and DBT-2Th) and comonomers (thiophene and bithiophene). Theoretical DFT calculations, electrochemical, and electrochromic properties were measured and compared. All polymeric precursor systems exhibited increasingly significant voltage drops (ΔV) of their reduction peaks with enhanced conjugation length. All conjugated polymers exhibited decreased HOMO-LUMO gaps, significantly improved electrochemical stability, and noteworthy color changes when transitioning from the oxidized to neutral state with increasing conjugation length.

## Data Availability Statement

The raw data supporting the conclusions of this article will be made available by the authors, without undue reservation.

## Author Contributions

KL and XL designed the experiments. KL, JH, CL, WT, QW, ZL, and YZ performed the experiments. KL, DW, and XL analyzed the experimental results. KL and XL wrote the manuscript. All authors commented on the manuscript. KL and XL supervised the project.

## Conflict of Interest

The authors declare that the research was conducted in the absence of any commercial or financial relationships that could be construed as a potential conflict of interest.
