# Supplementary material for: Electrochemical Synthesis and Electro-Optical Properties of Dibenzothiophene/Thiophene Conjugated Polymers With Stepwise Enhanced Conjugation Lengths
Source: Front Chem. 2020 Sep 8;8:819. doi: 10.3389/fchem.2020.00819 (PMC7505771; doi:10.3389/fchem.2020.00819)
Supplement: Supplementary file 1 [file Data_Sheet_1.DOC]

# Electrochemical Synthesis and Electro-optical Properties of Dibenzothiophene/Thiophene Conjugated Polymers with Stepwise Enhanced Conjugation Lengths

**Kaiwen Lin^1^*****, Caiting Li^2^, Wang Tao^3^, Jilong Huang^1^, Qinghua Wu^2^, Zijin Liu^2^, Yangfan Zhang^2^, Da Wang^3^, Xi Liu^2^***

^1^Department of Materials and Food, University of Electronic Science and Technology of China Zhongshan Institute, Zhongshan 528402, P.R. China

^2^School of Textile Materials and Engineering, Wuyi University, Jiangmen 529020, P. R. China

^3^School of Applied Physics and Materials, Wuyi University, Jiangmen 529020, P. R. China

*Correspondence:

Kaiwen Lin
[kevinlin1990@163.com](mailto:kevinlin1990@163.com)

Xi Liu
[liuxi_wyu@163.com](mailto:liuxi_wyu@163.com)

**EXPERIMENTAL SECTION**

Chemicals

*n*-Butyllithium (2.5 mol L^-1^ in hexane; Energy Chemical), chlorotributyltin (98%; Energy Chemical), Dibenzothiophene (DBT, 98%; Energy Chemical), thiophene (98%; Energy Chemical), bithiophene (98%; Energy Chemical), tetrabutylammonium hexafluorophosphate (Bu_4_NPF_6_, 98%; Energy Chemical), and tetrakis(triphenylphosphine)palladium(0) (Pd(PPh_3_)_4_, 99%; Energy Chemical) were stored at 4 ºC and used as received. Bromine and glacial acetic acid, chlorobenzene (analytical grade), chloroform (CHCl_3_, analytical grade), tetrahydrofuran (THF, analytical grade), dichloromethane (CH_2_Cl_2_, analytical grade), and acetonitrile (CH_3_CN, analytical grade) were purchased from Guangzhou Chemicals Co., Ltd. THF, CH_2_Cl_2_, and CH_3_CN were used after reflux distillation by sodium, phosphorus pentoxide, and calcium hydride, respectively.

Instrumentation

^1^H NMR and ^13^C NMR spectra were recorded on a Bruker AV 400 NMR spectrometer at ambient temperature. CDCl_3_ was used as the solvent and chemical shifts were recorded in ppm units with tetramethylsilane (TMS) as the internal standard. Density functional theoretical (DFT) calculations were performed at B3LYP/6-31G(d,p) level to study the molecular geometries and electron structures of the monomer.

**Synthesis of comonomers**

*Synthesis of* *2,8-dibromodibenzothiophene*

Bromine (3.1 mL, 60.5 mmol) was added dropwise to a mixture of DBT (5.0 g, 27.1 mmol) and chloroform (30.0 mL) at 0 ^o^C. Under nitrogen atmosphere the reaction mixture was stirred 12 h at room temperature. The crude product was filtered off and washed with methanol to isolate 2,8-dibromodibenzothiophene. The product was obtained as a white powder in 85% yield. mp: 218-221 °C; ^1^H NMR (400 MHz, CDCl_3_, ppm): *δ* 7.38 (d, *J* = 12.2 Hz, 2H), 7.52 (d, *J* = 7.8 Hz, 2H), 8.05 (d, *J* = 4.4 Hz, 2H).

*Synthesis of the stannylation of thiophene and bithiophene*

A solution of thiophene in dry THF was cooled to -78 °C and blanked by atomsphere (Ar) three times. *n*-BuLi (2.5 mol L^-1^ in hexane) was slowly added dropwise to the solution within 30 min. The mixture was stirred for 1.5 h at -78 °C and then warmed to -40 °C. Chlorotributyltin was added slowly to the solution, after that, the temperature was slowly warmed to room temperature. And the mixture was stirred at room temperature for 12 h under argon atmosphere. The residue was filtered, and then the solvent was removed under reduced pressure by rotary evaporation. The tributylstannane compound was used directly for the Stille coupling reaction without further purification.

Synthesis of 2-tributyl-stannyl-bithiophene was carried out in a similar manner to that of thiophene.

*Synthesis of 2,8-bis-(thiophen-2-yl)-dibenzothiophene (DBT-Th)*

DBT-Th was synthesized *via* Stille coupling reaction as described in Scheme 3. To a mixture solution of 2,8-dibromodibenzothiophene (1.0 g, 2.9 mmol) and tributyl(thiophen-2-yl)stannane (5.4 g, 14.5 mmol) in dry THF and Pd(PPh_3_)_4_ used as the catalyst was also added. The mixture was stirred magnetically at room temperature under nitrogen atmosphere. After 30 min, the mixture was heated to reflux with vigorous stirring for a further 24 h, and then concentrated under reduced pressure. Finally, column chromatography was used to purify the reaction mixture. The product was obtained as a milky white powder in 65% yield. ^1^H NMR (400 MHz, CDCl_3_, ppm): *δ* 7.20 (m, *J* = 8.2 Hz, 2H), 7.60 (d, *J* = 8.8 Hz, 2H), 7.75 (d, *J* = 4.4 Hz, 2H), 7.80 (m, *J* = 7.6 Hz, 2H), 8.05 (s, 1H), 8.07 (s, 1H), 8.84 (d, *J* = 4.0 Hz, 2H). ^13^C NMR (400 MHz, DMSO-*d*_6_, ppm): 143.73, 138.64, 136.07, 131.38, 128.95, 126.18, 125.51, 124.62, 124.14, 119.39.

*2,8-Bis-(bithiophen-2-yl)-dibenzothiophene (DBT-2Th)*

DBT-2Th was synthesised *via* Stille coupling reaction as described in Scheme 2. 2,8-Dibromodibenzothiophene (1.0 g, 2.9 mmol) and tributyl(bithiophene-2-yl) stannane (6.6 g, 14.5 mmol) were dissolved in dry THF (50 mL) with Pd(PPh_3_)_4_ (0.165 g) catalyst. The mixture was stirred under reflux condition for 24 h. The crude product was purified by column chromatography (eluent, dichloromethane:petrolem ether=1:2), obtaining a white powder in 65% yield. ^1^H NMR (400 MHz, CDCl_3_, ppm): δ 8.82 (s, 2H), 8.03 (d, 2H), 7.79 (d, 2H), 7.68 (d, 2H), 7.49 (d, 2H), 7.34 (d, 4H), 7.08 (d, 2H). ^13^C NMR (100 MHz, CDCl_3_, ppm): 143.06, 139.16, 137.47, 136.82, 135.87, 130.97, 127.85, 124.93, 124.68, 124.42, 123.96, 123.68, 123.26, 118.47.

**Electrochemistry**

All the electrochemical experiments and polymerization of monomers were performed in a one-compartment cell with the use of Model 263A potentiostat-galvanostat (EG&G Princeton Applied Research) under computer control. For electrochemical tests, the working and counter electrodes were both Pt wires with a diameter of 1 mm, while the reference electrode (RE) was Ag/AgCl. The Ag/AgCl RE was prepared electrochemically by chronoamperometry method at potential of 1.5 V for 100 s in hydrochloric acid (6 mol L^-1^) and calibrated against the SCE system. Bu_4_NPF_6_ (0.1 mol L^-1^) was used as electrolyte, dissolved in CH_2_Cl_2_ which was freshly distilled prior to its use. All the solutions were deaerated by a dry nitrogen stream and maintained under a slight overpressure through all the experiments. Polymer films were obtained electrochemically in potentiodynamic regime. After polymerization, the films were washed repeatedly with anhydrous MeCN to remove the electrolyte and monomer.

PDBT-Th was prepared from DBT-Th monomer (0.01 mol L^-1^) and Bu_4_NPF_6_ (0.1 mol L^-1^) in CH_2_Cl_2_ by electrochemical polymerization; PDBT-Th:Th was prepared from 0.005 mol L^-1^ DBT-Th and 0.005 mol L^-1^ thiophene monomer and Bu_4_NPF_6_ (0.1 mol L^-1^) in CH_2_Cl_2_; PDBT-2Th was prepared from DBT-2Th monomer (0.01 mol L^-1^) and Bu_4_NPF_6_ (0.1 mol L^-1^) in CH_2_Cl_2_; PDBT-Th:2Th was prepared from 0.005 mol L^-1^ DBT-Th and 0.005 mol L^-1^ bithiophene monomer and Bu_4_NPF_6_ (0.1 mol L^-1^) in CH_2_Cl_2_; PDBT-2Th:Th was prepared from 0.005 mol L^-1^ DBT-2Th and 0.005 mol L^-1^ thiophene monomer and Bu_4_NPF_6_ (0.1 mol L^-1^) in CH_2_Cl_2_; PDBT-2Th:2Th was prepared from 0.005 mol L^-1^ DBT-2Th and 0.005 mol L^-1^ bithiophene monomer and Bu_4_NPF_6_ (0.1 mol L^-1^) in CH_2_Cl_2_.

The cyclic voltammetry was employed to evaluate the HOMO levels. LUMO levels was evaluated by *E*_g_ and HOMO. The empirical equations were as follow:^1^

HOMO = - (*E*_ox_+4.8) eV (1)

LUMO = - (*E*_red_+4.8) eV (2)

where *E*_ox_ and *E*_red_ are the onset oxidation and reduction potentials of polymers, respectively.

**Electrochromic experiments**

All the electrochemical experiments and polymerization of monomers were performed in a one-compartment cell with the use of Model 263A potentiostat-galvanostat (EG&G Princeton Applied Research) under computer control. For electrochemical tests, the working and counter electrodes were both Pt wires with a diameter of 1 mm, while the reference electrode (RE) was Ag/AgCl. The Ag/AgCl RE was prepared electrochemically by chronoamperometry method at potential of 1.5 V for 100 s in hydrochloric acid (6 mol L^-1^) and calibrated against the SCE system. Bu_4_NPF_6_ (0.1 mol L^-1^) was used as electrolyte, dissolved in CH_2_Cl_2_ which was freshly distilled prior to its use. All the solutions were deaerated by a dry nitrogen stream and maintained under a slight overpressure through all the experiments. Polymer films were obtained electrochemically in potentiodynamic regime. After polymerization, the films were washed repeatedly with anhydrous CH_3_CN to remove the electrolyte and monomer.

Spectroelectrochemistry and kinetic studies were recorded on a Specord 200 plus (Analytik Jena) spectrophotometer and the potentials were cont rolled using Versa STAT 3 (Princeton Applied Research). The spectroelectrochemical cell consisted of a quartz cell, an Ag/AgCl electrode as reference electrode, a Pt wire as counter electrode, and an indium tin oxide (ITO) coated glass as the transparent working electrode. All measurements were carried out in CH_3_CN containing Bu_4_NPF_6_ (0.1 mol L^-1^).

Optical band gap (*E*_g,opt_) is calculated according to the onset absorption of π–π* transition (*λ*_onset_) with the following formula:

*E*_g,opt_ = 1240/*λ*_onset_ (3)


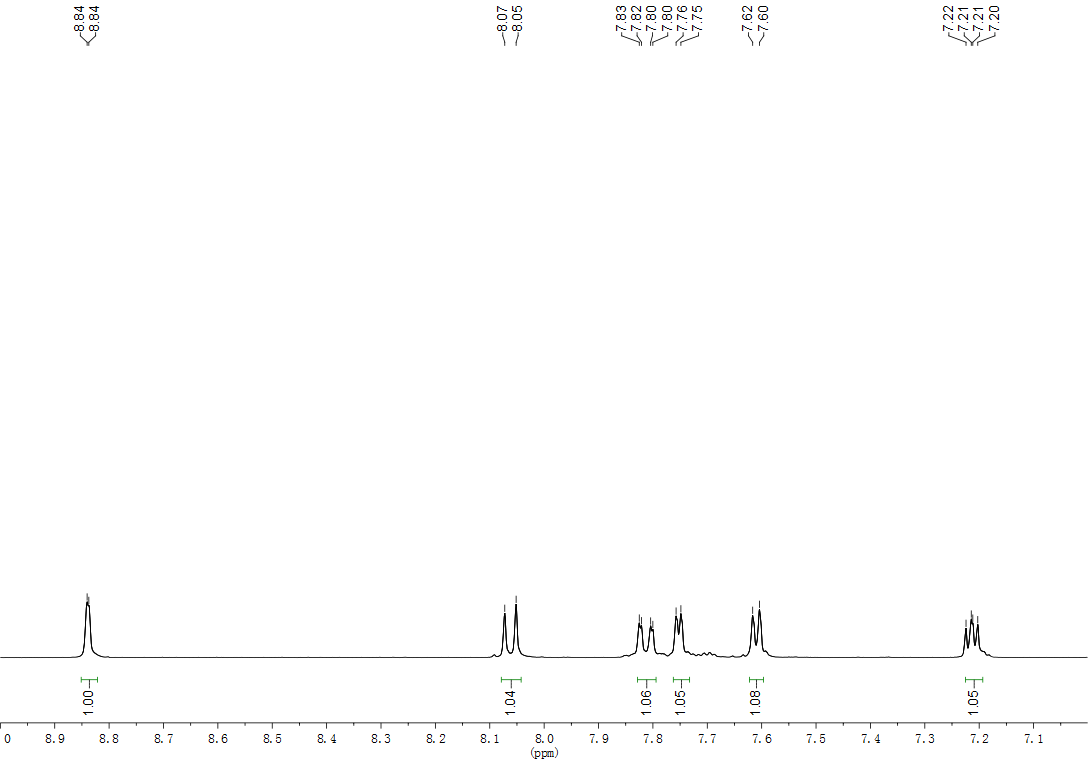


**FIGURE S1** ^1^H NMR spectrum of DBT-Th.


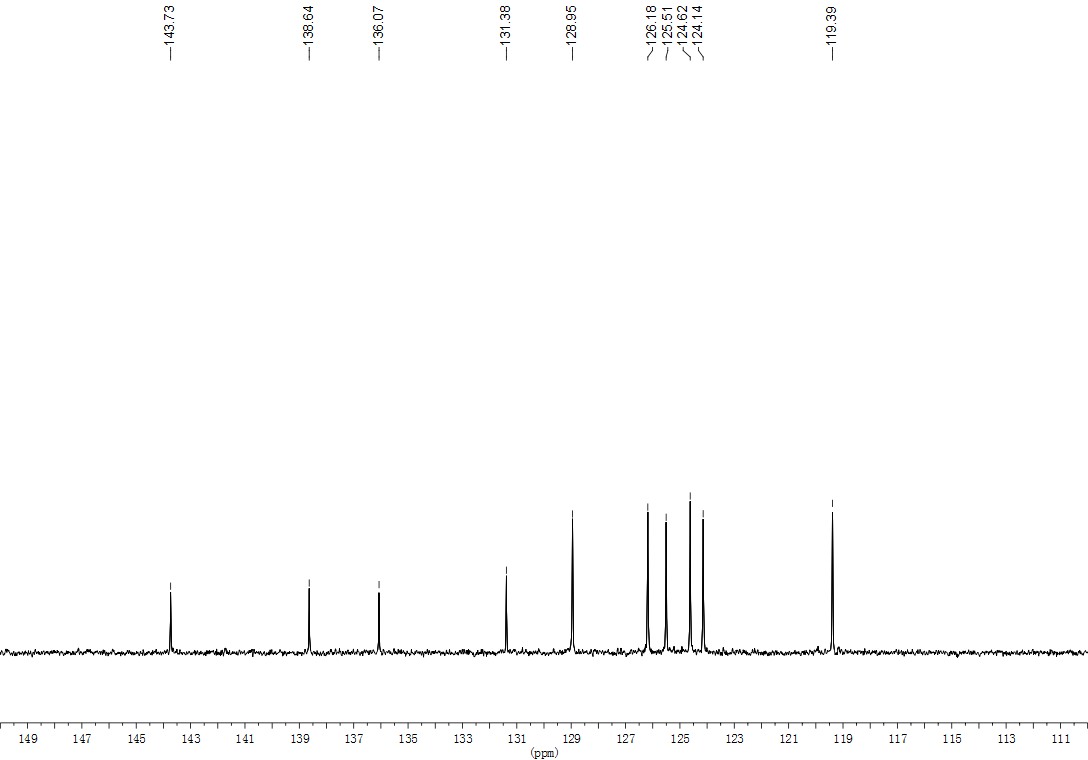


**FIGURE S2** ^13^C NMR spectrum of DBT-Th.


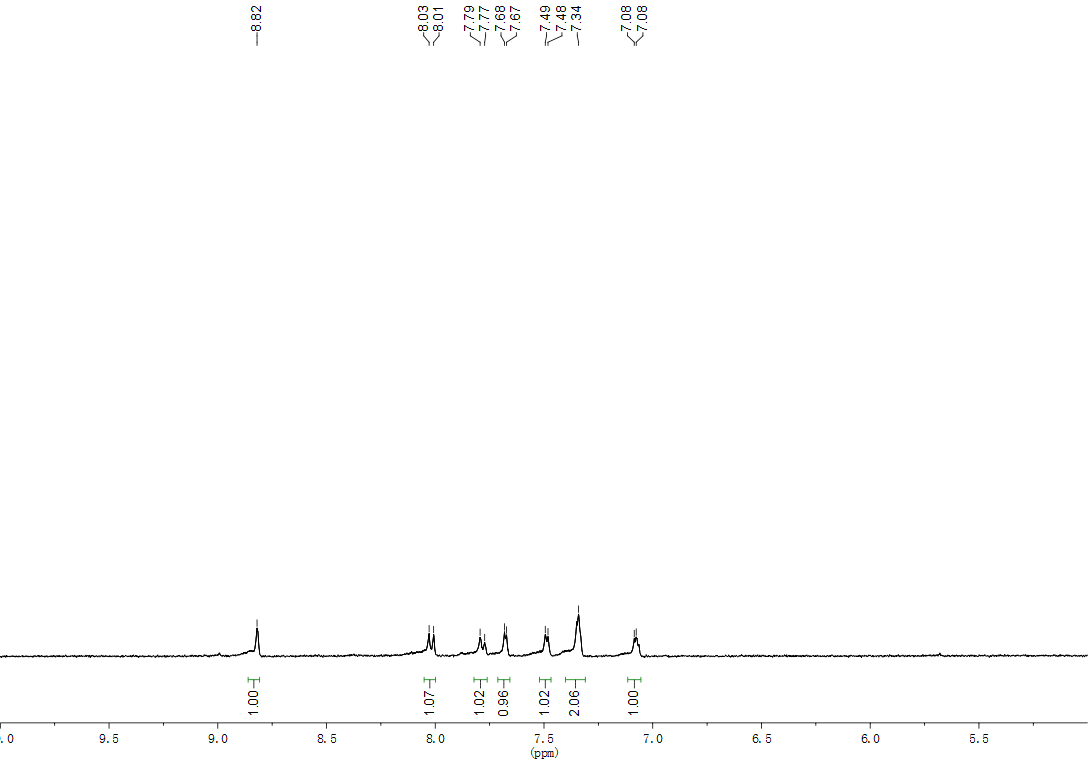


**FIGURE S3** ^1^H NMR spectrum of DBT-2Th.


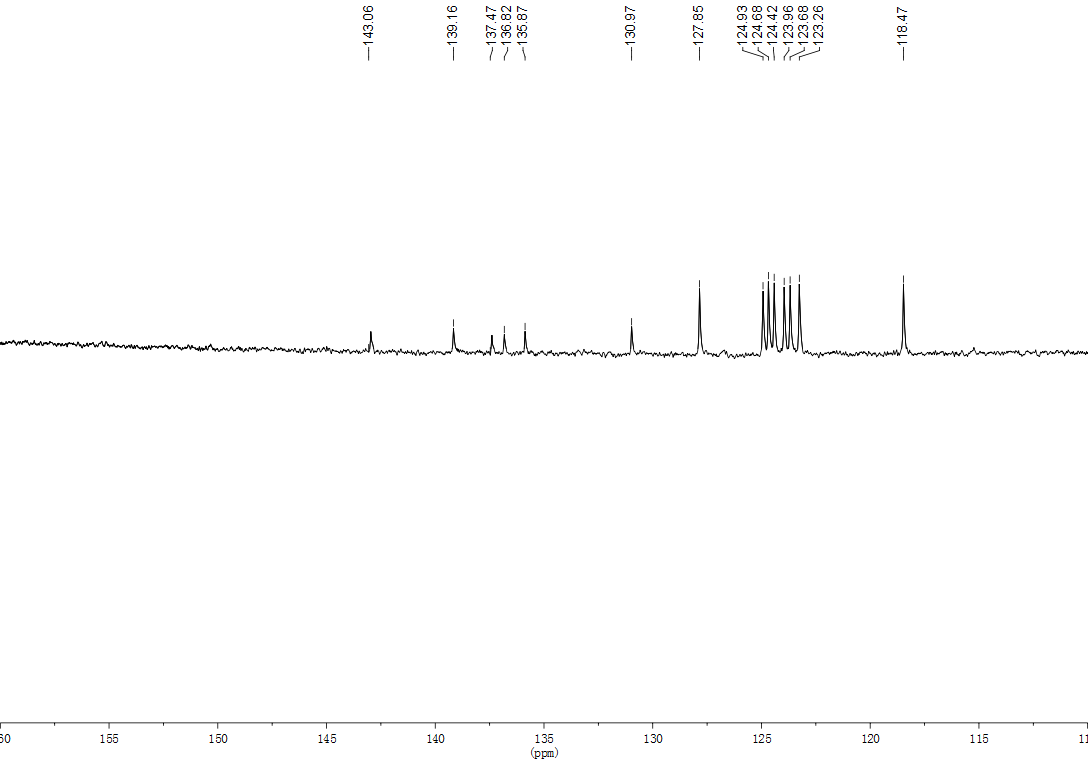


**FIGURE S4** ^13^C NMR spectrum of DBT-2Th.

**
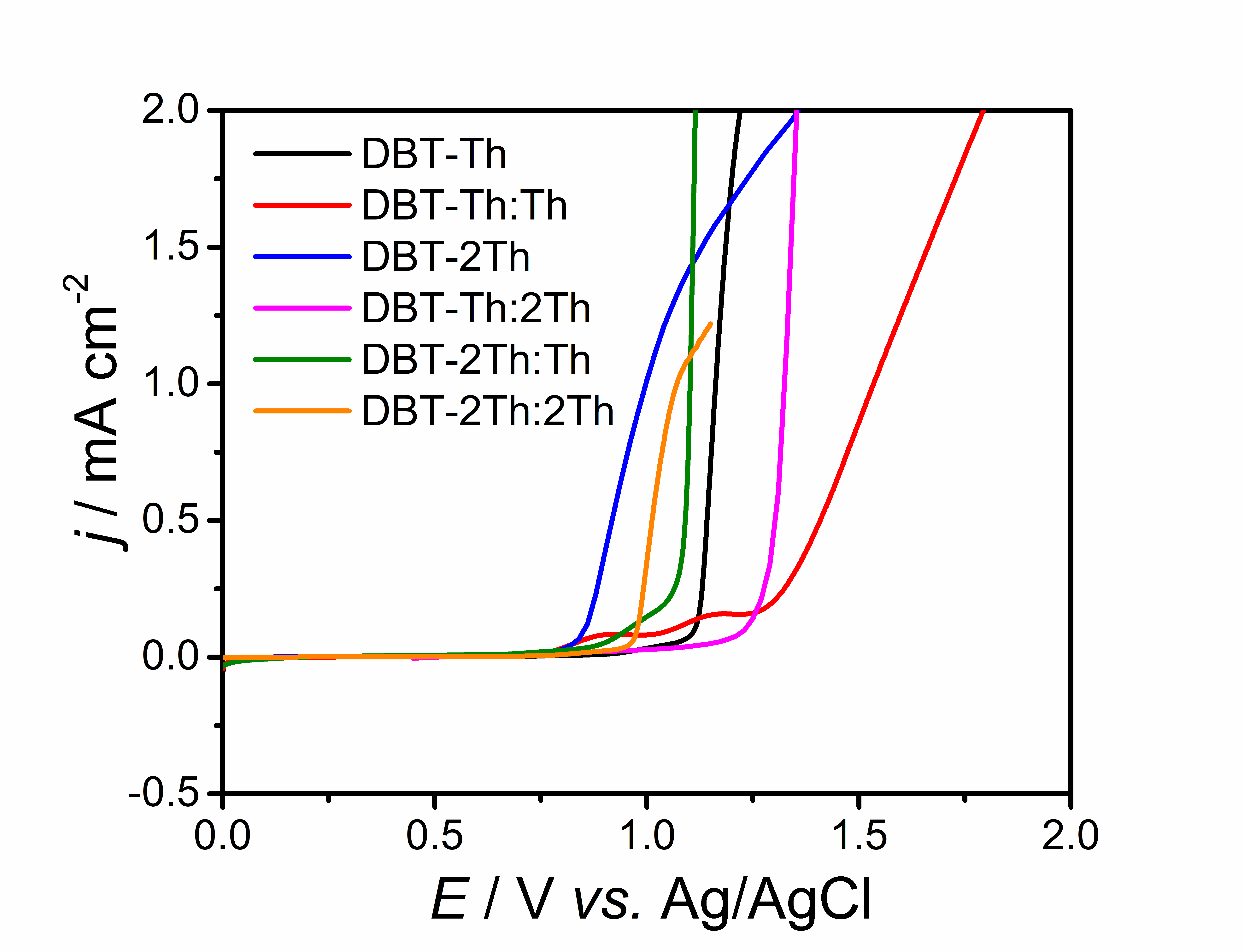
**

**FIGURE S5** Anodic polarization curves of 0.01 mol L^-1^ monomers and comonomers in CH_2_Cl_2_-Bu_4_NPF_6_ (0.1 mol L^-1^). Potential scan rate: 50 mV s^-1^. **
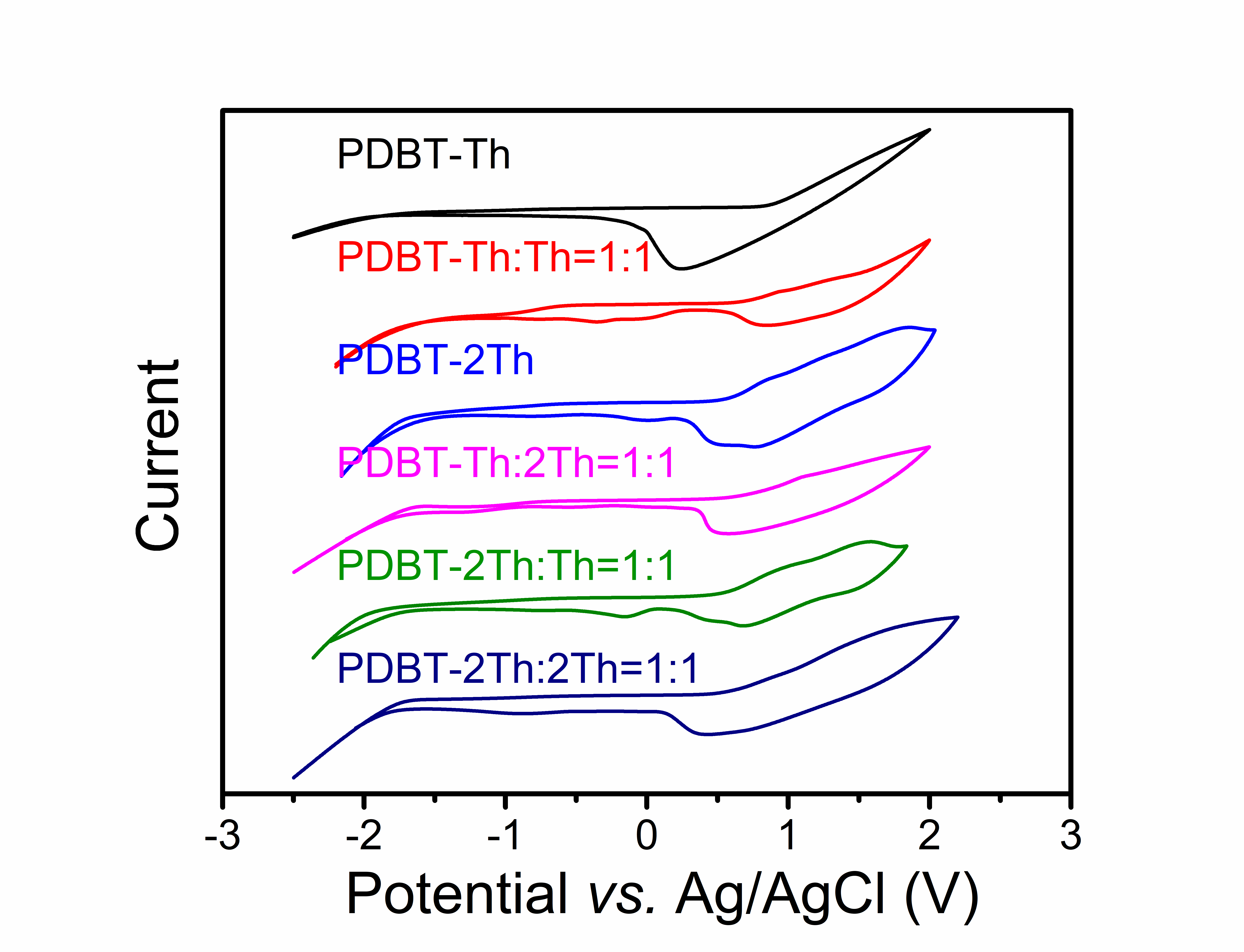
**

**FIGURE S6** Cyclic voltammetry of polymers.


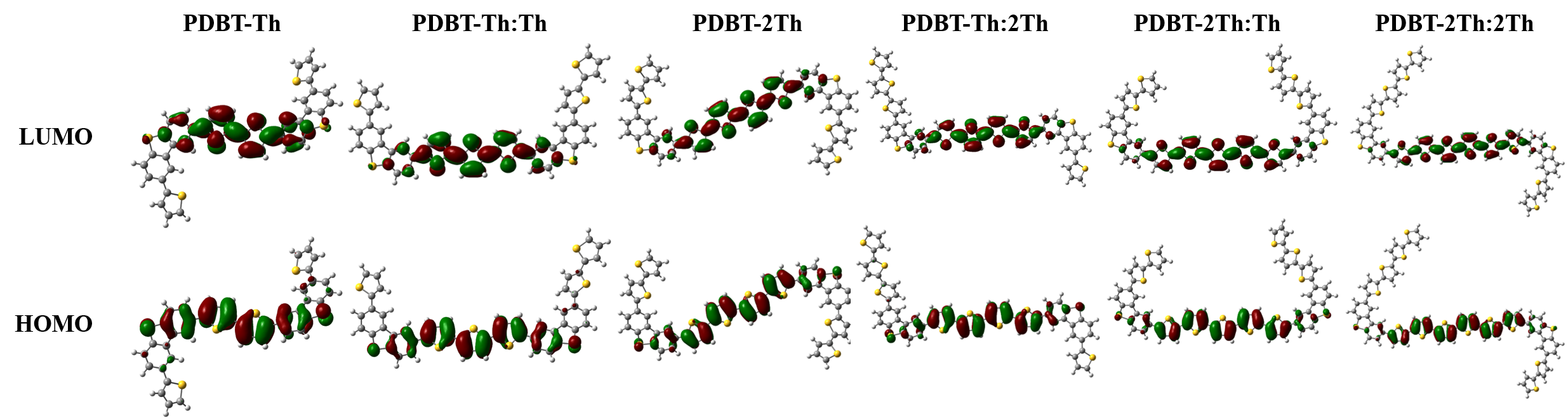


**FIGURE S7** The theoretical calculated HOMO/LUMO energy levels of polymers (simplified by two repeating units) obtained from DFT by Gaussian 09 at the B3LYP/6-31G(d,p) level.

Reference

1 Sun, Y., Chien, S. C., Yip, H. L., Zhang, Y., Chen, K. S., Zeigler, D. F., Chen, F. C., Lin, B., and Jen, A. K. Y. (2011). High-mobility Low-bandgap Conjugated Copolymers Based on Indacenodithiophene and Thiadiazolo[3,4-c]pyridine Units for Thin Film Transistor and Photovoltaic Applications. *J. Mater. Chem.* 21, 13247–13255. doi: 10.1039/c1jm11564b
